# Design of typical genes for heterologous gene expression

**DOI:** 10.1038/s41598-022-13089-1

**Published:** 2022-06-10

**Authors:** Dominic Simm, Blagovesta Popova, Gerhard H. Braus, Stephan Waack, Martin Kollmar

**Affiliations:** 1 Theoretical Computer Science and Algorithmic Methods, Institute of Computer Science, Georg-August-University Göttingen, Göttingen, Germany; 2 Group Systems Biology of Motor Proteins, Department of NMR-Based Structural Biology, Max-Planck-Institute for Biophysical Chemistry, Göttingen, Germany; 3 Molecular Microbiology and Genetics, Institute for Microbiology and Genetics and Göttingen Center for Molecular Biosciences (GZMB), Georg-August-University Göttingen, Göttingen, Germany

**Keywords:** Synthetic biology, Bioinformatics

## Abstract

Heterologous protein expression is an important method for analysing cellular functions of proteins, in genetic circuit engineering and in overexpressing proteins for biopharmaceutical applications and structural biology research. The degeneracy of the genetic code, which enables a single protein to be encoded by a multitude of synonymous gene sequences, plays an important role in regulating protein expression, but substantial uncertainty exists concerning the details of this phenomenon. Here we analyse the influence of a profiled codon usage adaptation approach on protein expression levels in the eukaryotic model organism *Saccharomyces cerevisiae*. We selected green fluorescent protein (GFP) and human α-synuclein (αSyn) as representatives for stable and intrinsically disordered proteins and representing a benchmark and a challenging test case. A new approach was implemented to design typical genes resembling the codon usage of any subset of endogenous genes. Using this approach, synthetic genes for GFP and αSyn were generated, heterologously expressed and evaluated in yeast. We demonstrate that GFP is expressed at high levels, and that the toxic αSyn can be adapted to endogenous, low-level expression. The new software is publicly available as a web-application for performing host-specific protein adaptations to a set of the most commonly used model organisms (https://odysseus.motorprotein.de).

## Introduction

Modifying gene sequence is an important step when generating sequences for homologous and heterologous protein expression^1–5^. This allows, for example, to investigate functions of homologous proteins^6^, to synthetically construct genetic circuits^7–9^, or to overexpress proteins for biopharmaceutical applications^10^ and structural biology research^11^. These types of experiments have on the one hand highly profited from the exponentially accumulating sequence information from genome and transcriptome sequencing projects. On the other hand, the considerable increase in speed and decrease in costs for synthetic gene synthesis provides a convenient way to obtain physical genes encoding the desired proteins.

The genetic code redundancy allows adjusting gene sequences without changing the protein sequences. This principle is used for a long time for practical aspects such as facilitating cloning by adding or removing restriction sites or by removing internal Shine–Dalgarno consensus sequences. Here, just one or a few codons are altered. Adjusting all codons of a gene to a certain codon usage frequency is often referred to as codon optimization^12,13^. In synthetic biology the optimization goal is mostly increased protein expression, whereas in many other biological applications overexpressed proteins might generate unwanted effects and adjustment to the codon usage frequency of lowly expressed proteins might be preferred. Most gene design tools optimize the codon adaptation index (CAI)^14^, which is the deviation of a protein coding sequence from a set of reference genes and ranges from 0 to 1. Very simply, the CAI of a gene is optimized to perfection if only the most used codons of the reference gene sets are used. For most applications the reference gene sets just consist of a few to a few dozen genes, of which most encode ribosomal proteins^14,15^.

Instead of this rather statistical approach that evaluates gene sequences by codon counting, gene design can be driven by biochemical observations and deeper understanding of the ribosomal translation. In the protein biosynthesis process, the simultaneous presence of two tRNAs in the A and P positions of the ribosome is necessary for the formation of a peptide bond^16–18^. Due to steric reasons not all combinations of codons and tRNAs are equally compatible to the ribosome surface, which means that certain codon pairs are processed more efficiently than others. If this were the case it would be expected that the observed frequency of occurrence of a codon pair would significantly deviate from its statistically predicted mean value. Analysing 237 protein coding genes from *E. coli* demonstrated that some codon pairs were overrepresented while others were underrepresented in comparison with the theoretical predicted means^19^. This study has later been extended to all protein coding genes of the *E. coli* genome^20^ and also to several hundred organisms from all three domains of life^21^. The phenomenon of the non-random utilization of codon pairs is called the ‘codon context’ and is assumed to correlate with the translation elongation rate in a way that rare codon pairs decrease the rate^22^. Only few gene design software use codon context information^23–25^.

Here, we developed a software to design “typical genes”. Typical genes are not optimized against parameters such as CAI or codon usage but are intended to show a similar codon distribution as compared to a reference gene set, which can be a selection of highly or lowly expressed genes or a selection of genes having a similar cellular context such as transmembrane or cytoskeletal proteins. Because many studies showed that heterologous proteins can strongly be overexpressed although they have a counterintuitively wrong codon usage, we developed a formalism to invert a selected codon usage. The new design algorithm was tested by designing typical genes for green fluorescent protein (GFP^26^) and human α-synuclein (αSyn^27^) and evaluating their expression in the unicellular budding yeast *Saccharomyces cerevisiae*.

## Materials and methods

### Model for generating typical genes

**Figure 1:**
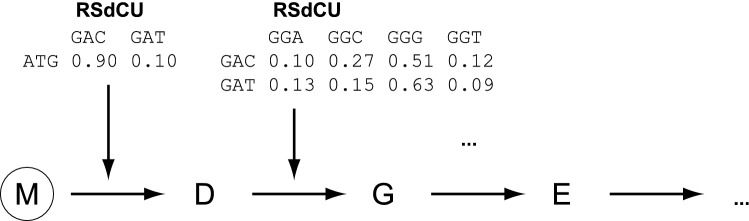
Example of a Markov chain. For a protein sequence starting with M-D-G-E a typical gene sequence will be designed. The RSdCU frequencies are computed based on the set of selected sequences, which could be all genes of a species, the sub-section of the 10% most highly expressed genes of a species, the selection of all genes coding for trans-membrane proteins of a species, or any other user-specified set of genes. All frequencies are normalized within each codon box. The Markov chain is built by using the RSdCU for the transition/emission probabilities.

Given a protein sequence a set of typical genes is generated using a Markov chain model. The Markov chain is built by using the relative synonymous di-codon usage frequencies (*RSdCU*) for the transition/emission probabilities (Fig. 1). The relative codon usage refers to the usage of a codon with respect to all 61 sense codons, the relative synonymous codon usage refers to the usage of a codon within the set of codons coding for the same amino acid. The relative di-codon usage refers to the frequency of each set of two neighbouring codons. The RSdCU is defined here as the relative synonymous codon usage of the second codon of a di-codon with respect to all codons coding for the same second amino acid. All frequencies are normalized within each codon box. The reference for all the codon usage metrics is the codon usage within a set of genes. Usually, for heterologous gene expression the set of genes is taken from the host organism, to which the gene sequence obtained from another species should be adapted. But in principle any set of genes can be chosen. By allowing the user to define a selection of genes, the gene of interest can be adapted to the codon usage of any subset of genes of a host organism, e.g. the most highly expressed genes, genes involved in metabolism, or genes coding for transmembrane proteins. To speed up the process of generating the RSdCU matrices, the RSdCU is pre-calculated from data for a number of pre-defined subsets of genes such as the selection of the highest expressed yeast genes.

### Collecting and processing codon usage and protein abundance data

Protein abundance datasets were obtained from the publicly available PaxDB database^28^. PaxDB provides unified protein quantification data with proteome-wide coverage derived from biophysical and mass spectrometry studies for a broad range of organisms. The associated coding sequences (CDS) were collected via the Entrez-API^29^ from the National Center of Biotechnology Information (NCBI) in May 2018. The downloaded sequences were checked, and partial and invalid gene sequences as well as sequences with obvious problems (i.e. discontinued genes, internal reading-frame shifts, in-frame stop codons) were removed.

The protein abundance data allows sampling of the proteins by cellular protein abundance levels. The abundance is given in ‘ppm’ (parts per million) and varies over several powers of ten. This means that by simply counting the corresponding codons in every subset of proteins the corresponding codons of the lowest expressed proteins would get the same weight as the corresponding codons of the highest expressed proteins, although their expression level varies considerably. To accomplish for the different abundance level of the proteins, each codon is therefore multiplied with the protein abundance resulting in the weighted codon-usage parameter-set *weighted-RSdCU*.

The annotation of the sequence data (e.g. cellular localization, biomolecular function) and the reference to the protein abundance data (from PacDB) allows the generation of organism-specific and gene set-specific RSdCU computations for the Markov chain model. For example, based on the protein abundance information just the 50 highest expressed proteins of an organism could be selected, or the 2000 least expressed proteins. In other use cases, for example, the ATPases, membrane proteins, or cytoskeletal proteins of an organism could be selected to generate the RSdCU matrix. This approach allows the flexible computation of the RSdCU for a diverse set of organisms as target hosts, for a set of proteins with similar expression level, and for a selected set of proteins with similar cellular function.

### Inverting the codon usage

While we compared the codon usage of many non-yeast genes with the codon usage of yeast, we observed that the codon usage of the non-yeast genes is often different, different not by showing a random different codon usage but by kind of “inverting” the difference of the codon usage of the highly expressed yeast genes compared to the yeast codon usage. For example, let the yeast codon usage of CAA and CAG be 0.68 and 0.32, respectively. Selecting only the highest expressed genes the codon usage of CAA and CAG were 0.93 and 0.07, respectively. Thus, in highly expressed genes, the codon usage of CAA is increased by 0.25 while the codon usage of CAG decreases by 0.25. “Switching” the codon usage would result in a dramatic change of the codon usage to 0.07 for CAA and 0.93 for CAG, which is not observed. Rather, the codon usage of the non-yeast genes resembles a scheme, where the difference of 0.25 between highly expressed genes and yeast codon usage is inverted, resulting in codon usage of 0.43 and 0.57 for CAA and CAG, respectively. Therefore, we here coin the term “inverted codon usage” for codon usages, which are generated by reversing the codon usage frequencies within each set of synonymous codons with respect to a reference codon usage. As reference, we here used the genome-wide frequency of each codon as described in the example above. With this reference the inversion of the codon usage results in patterns of typical codon frequency distributions, and not in rather artificial codon usages as generated by “switching” the codon usage within synonymous codons. The inversion thus represents kind of a mirror operation on the values of the genome-wide frequencies used as reference. To make the computation of the inversion fail-safe, the inverted codon usage of the respective codon is set to 0.05 or 0.95, respectively, in case the inversion would result in a negative RCU or an RCU bigger than 1 (this can only happen when extremely rare codons in the reference are the most prevalent in the set of selected sequences and vice versa). The difference between the computed inverted codon usage and the 0.05 or 0.95 setting is then proportionally subtracted from or added to the frequencies of the other codons of the codon box. For generating the RSdCU matrix, the RCUs for each codon box are inverted in both dimensions of the matrix, e.g. first inverting the codon usage of the first codon of the di-codon and then inverting the codon usage of the second codon of the di-codon (Supplementary Fig. S5).

### Post-processing and filtering the initial set of typical genes

The generated sequences might contain patterns unfavourable for subsequent experimental work (e.g. presence of enzyme restriction sites) and/or patterns unfavourable for translation initiation (e.g. strong base pairing at the 5′-end of the mRNAs). Such patterns are determined in several post-processing steps. Instead of modifying the respective sequences to remove the patterns, which would lead to local deviations from the RSdCU, sequences containing the patterns are removed and further typical sequences generated. To allow filtering for restriction sites wanted or eliminated for cloning and control the respective sequence pattern information has been collected from the Restriction Enzyme Database (REBASE^30^). To allow filtering for unfavourable base pairings, RNAfold from the ViennaRNA Package^31^ was integrated for prediction of mRNA stability.

### Software implementation

**Figure 2:**
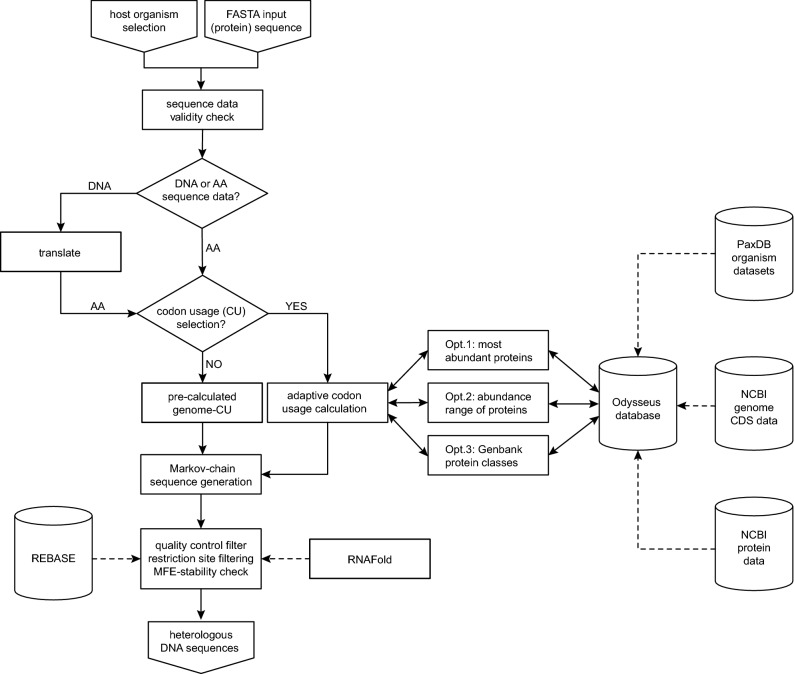
Odysseus flowchart. The input for the process (top of the scheme) are a sequence (protein or DNA) in FASTA format and the selection of the host organism for which the gene will be designed. The resulting DNA sequence is the output of the process (bottom of the scheme). Computations during the process are represented by boxes, databases by cylinders, decisions by diamonds and the direction of data flow by arrows. Data input from external databases and computations with external software are represented by dotted lines.

The gene reconstruction algorithm is written in Python 2.7 and available as software termed Odysseus (Fig. 2). The software can be used via a web interface at http://odysseus.motorprotein.de, and obtained from GitHub at https://github.com/dsimm/Odysseus for local installation and use. Odysseus requires input of a protein or cDNA sequence, the latter being translated subsequently. Next, the web interface allows selecting a host-organism and adjusting model-parameters such as selection of subsets of proteins. Typical genes are then generated using pre-computed or dynamically assembled codon-usage profiles. Pre-computed profiles are available for multiple organisms based on various expression level ranges, which were termed ‘Low’, ‘Mid’ and ‘High’. If the user filters proteins for their cellular function or through a systematic selection using the annotated PaxDB expression information the profile-dataset is computed dynamically. This is the more time-consuming option and should only be considered, if there is need for a more specific adaptation of the model parameters of the Markov chain to characteristic protein groups of the targeted host organism.

### Plasmid construction, yeast strains, transformation and growth conditions

Plasmids and *Saccharomyces cerevisiae* strains are listed in Tables 1 and 2. DNA coding sequences were synthesized by Life Technologies, Darmstadt, Germany. The synthetic DNA fragments were cloned into the SmaI site of the integrative plasmid pRS306 using GENEART Seamless cloning and assembly kit (Life Technologies, Darmstadt, Germany). All constructs were verified by DNA sequencing. The *GAL1-SNCA* or *GAL1-GFP* sequences were integrated into the mutated *ura3-1* or *trp1-1* locus of *S. cerevisiae* W303-1A strain using an intact *URA3* or *TRP1* gene on the corresponding integrative plasmid for selection. The number of the integrated copies was determined by Southern hybridization as described previously^32^.

**Table 1:** Plasmids used in this study.

Plasmid	Description	Source
pRS306	pRS306*-GAL1-Promoter, CYC1-Terminator, URA3, integrative, pUC origin, Amp*^*R*^	^36^
pME4859	pRS306-*GFP* (low-expression-weighted*; gene1*)	This study
pME4860	pRS306-*GFP* (high-expression-weighted*; gene2*)	This study
pME4861	pRS306-*GFP* (high-expression-weighted-inverted*; gene3*)	This study
pME4853	pRS306*-SCNA (low expression; gene4)*	This study
pME4854	pRS306-*SCNA (middle expression; gene5)*	This study
pME4855	pRS306-*SCNA (high expression; gene6)*	This study
pME4856	pRS306-*SCNA* (low-expression-weighted*; gene7*)	This study
pME4857	pRS306-*SCNA* (high-expression-weighted*; gene8*)	This study
pME4858	pRS306-*SCNA* (high-expression-weighted-inverted*; gene9*)	This study

**Table 2:** Yeast strains used in this study.

Strain	Genotype	Source
W303-1A	*MATa; ura3-1; trp1-1; leu2-3_112; his3-11; ade2-1; can1-100*	EUROSCARF
RH3771	W303 containing 1 genomic copy *GAL1::GFP (low expression-weighted; gene1)* in *ura3* locus	This study
RH3772	W303 containing 2 genomic copy *GAL1::GFP (low expression-weighted; gene1)* in *ura3* locus	This study
RH3773	W303 containing 3 genomic copy *GAL1::GFP (low expression-weighted; gene1)* in *ura3* locus	This study
RH3774	W303 containing 1 genomic copy *GAL1::GFP (high expression-weighted; gene2)* in *ura3* locus	This study
RH3775	W303 containing 2 genomic copy *GAL1::GFP (high expression-weighted; gene2)* in *ura3* locus	This study
RH3776	W303 containing 3 genomic copy *GAL1::GFP (high expression-weighted; gene2)* in *ura3* locus	This study
RH3777	W303 containing 1 genomic copy *GAL1::GFP (high expression-weighted-inverted; gene3)* in *ura3* locus	This study
RH3778	W303 containing 2 genomic copy *GAL1::GFP (high expression-weighted-inverted; gene3)* in *ura3* locus	This study
RH3779	W303 containing 3 genomic copy *GAL1::GFP (high expression-weighted-inverted; gene3)* in *ura3* locus	This study
RH3756	W303 containing 1 genomic copy *GAL1::SNCA (low expression; gene4)* in *ura3* locus	This study
RH3757	W303 containing 2 genomic copy *GAL1::SNCA (low expression; gene4)* in *ura3* locus	This study
RH3758	W303 containing 1 genomic copy *GAL1::SNCA (middle expression; gene5)* in *ura3* locus	This study
RH3759	W303 containing 2 genomic copy *GAL1::SNCA (middle expression; gene5)* in *ura3* locus	This study
RH3760	W303 containing 1 genomic copy *GAL1::SNCA (high expression; gene6)* in *ura3* locus	This study
RH3761	W303 containing 2 genomic copy *GAL1::SNCA (high expression; gene6)* in *ura3* locus	This study
RH3762	W303 containing 1 genomic copy *GAL1::SNCA (low expression-weighted; gene7)* in *ura3* locus	This study
RH3763	W303 containing 2 genomic copy *GAL1::SNCA (low expression-weighted; gene7)* in *ura3* locus	This study
RH3764	W303 containing 3 genomic copy *GAL1::SNCA (low expression-weighted; gene7)* in *ura3* locus	This study
RH3765	W303 containing 1 genomic copy *GAL1::SNCA (high expression-weighted; gene8)* in *ura3* locus	This study
RH3766	W303 containing 2 genomic copy *GAL1::SNCA (high expression-weighted; gene8)* in *ura3* locus	This study
RH3767	W303 containing 3 genomic copy *GAL1::SNCA (high expression-weighted; gene8)* in *ura3* locus	This study
RH3768	W303 containing 1 genomic copy *GAL1::SNCA (high expression-weighted-inverted; gene9)* in *ura3* locus	This study
RH3769	W303 containing 2 genomic copy *GAL1::SNCA (high expression-weighted-inverted; gene9)* in *ura3* locus	This study
RH3770	W303 containing 3 genomic copy *GAL1::SNCA (high expression-weighted-inverted; gene9)* in *ura3* locus	This study
RH3780	W303 containing 1 genomic copies *GAL1::SNCA (human)* in *trp1* locus	This study
RH3781	W303 containing 2 genomic copies *GAL1::SNCA (human)* in *trp1* locus	This study
RH3465	W303 containing 1 genomic copy *GAL1::GFP* in *ura3* locus	^32^
RH3466	W303 containing 1 genomic copy *GAL1::SNCA (human)-GFP* in *ura3* locus	^32^
RH3467	W303 containing 2 genomic copies *GAL1::SNCA (human)-GFP* in *ura3* locus	^32^

*Saccharomyces cerevisiae* strain W303-1A was used for transformations performed by standard lithium acetate protocol^33^. All strains were grown in Synthetic complete (SC) medium^34^ lacking the corresponding marker and supplemented with 2% raffinose or 2% glucose. αSyn or GFP expression was induced by shifting yeast cells cultivated overnight in raffinose to 2% galactose-containing medium (OD_600_ = 0.1).

### Spotting assay

For growth test on solid medium, yeast cells were pre-grown in SC-selection medium containing 2% raffinose to mid-log phase. Cells were normalized to equal densities, serially diluted tenfold starting with an OD_600_ of 0.1, and spotted on SC-plates containing either 2% glucose or 2% galactose. After three days incubation the plates were photographed.

### Immunoblotting

Yeast cells harboring αSyn or GFP-encoding genes were pre-grown at 30 °C in SC-selection medium containing 2% raffinose. Cells were transferred to SC medium containing 2% galactose at OD_600_ = 0.1 to induce the *GAL1* promoter for 6 h. Total protein extracts were prepared as described^35^ and the protein concentrations were determined with a Bradford assay. Equal amounts from each protein sample were subjected to 12% SDS–polyacrylamide gel electrophoresis and transferred to a nitrocellulose membrane. Membranes were probed with αSyn rabbit polyclonal antibody (Santa Cruz Biotechnology, USA) or GFP rat monoclonal antibody (Chromotek, Germany). GAPDH mouse monoclonal antibody (Thermo Fisher Scientific, USA) was used as loading controls. Pixel density values for Western quantification were obtained from TIFF files generated from digitized X-ray films (KODAK) and analyzed with the ImageJ software (NIH, Bethesda, USA). Before comparison, sample density values were normalized to the corresponding loading control. We have included images of all original full-length membranes in the supplementary files. The uncropped full length images are labelled as in the main text and are presented as originally scanned. The membrane edges are not visible due to short exposure times.

### Fluorescence microscopy and quantifications

Yeast cells harboring GFP were grown in SC-selective medium containing 2% raffinose overnight, and transferred to 2% galactose containing medium for induction of GFP expression for 6 h. Fluorescent images were obtained with Zeiss Observer. The Z1 microscope (Zeiss) was equipped with a CSU-X1 A1 confocal scanner unit (YOKOGAWA), QuantEM:512SC digital camera (Photometrics) and SlideBook 6.0 software package (Intelligent Imaging Innovations). At least 100 cells were measured per strain and per experiment for quantification of fluorescent intensities.

### RNA isolation and quantitative real-time PCR

Total RNA was isolated using the ‘High Pure RNA Isolation Kit’ (Roche Diagnostics GmbH, Mannheim, Germany) from yeast cells that were grown in SC-selective medium containing 2% galactose for induction of *GAL1* promoter for 6 h. cDNA synthesis was performed in duplicates for each sample using 0.8 µg RNA and the QuantiTect Reverse Transcription Kit (Qiagen, Hilden, Germany) according to the manufacturer’s instructions. Amplification was performed with CFX Connect Real-Time System (Bio-Rad) with MESA GREEN qPCR MasterMix Plus for SYBR Assay (Eurogentec) and analyzed in three technical repeats. The expression of Histone h2A was used as reference.

## Results and discussion

Most approaches to computationally reconstruct genes from heterologous protein sequences concentrate on optimizing the usage of so-called preferred codons (also called more frequent codons, optimal codons, or major codons) with preference usually being regarded as present in highly expressed proteins. Because there is no definition for highly expressed protein and the set of most strongly expressed proteins likely differs from species to species, multiple ways have been suggested to derive the subset of preferred codons. According to one of the earliest approaches developed in the 1980s the preferred codon is assigned to that codon of a codon box that is most frequently used across ribosomal genes. At that time this was likely the best approach given the limit in available sequences and expression data. However, while highly expressed and translated, ribosomal proteins are not representative for the cellular proteome. In another method, the overall codon bias of each gene is determined and those codons, whose frequencies within the gene most significantly positively correlate with the bias, are assigned as preferred codons^1^.

### tRNA abundance does not correlate with codon usage in many codon boxes

In another approach, a codon is termed preferred codon if it is recognized by the tRNA that has either the highest copy number in the genome or the highest cellular expression level. This method of course does not work for synonymous codons that are recognized by tRNAs with equal copy numbers. In addition, decoding is highly redundant and wobble decoding is often as efficient as decoding of cognate codons^37–39^. In many codon boxes that codon, for which there are the most tRNA gene copies, is more used in highly expressed genes than any of the other synonymous codons of the box. In contrast, the usage of the wobble decoded GGU- and UGU-codons, for which cognate tRNAs are absent in all yeast genomes available to date^40^, is considerably increased in the highly expressed yeast genes. Also the frequency of, for example, the AUC-, ACC-, and GUC-codons, for which cognate tRNAs also do not exist, increases more strongly in those genes whose products are detectably present in the proteomes compared to the usage frequency of synonymous codons with cognate tRNAs. Similarly, the usage frequency of UUG triplicates in highly expressed genes while the frequency of UUA decreases although the number of cognate tRNAs is very similar (ten cognate tRNAs compared to seven, respectively). Thus, total tRNA abundance does not correlate in all codon boxes with codon usage in highly expressed genes. We therefore refrained from designing genes based on tRNA presence and abundance data.

### Protein abundance as reference for codon usage bias

**Figure 3:**
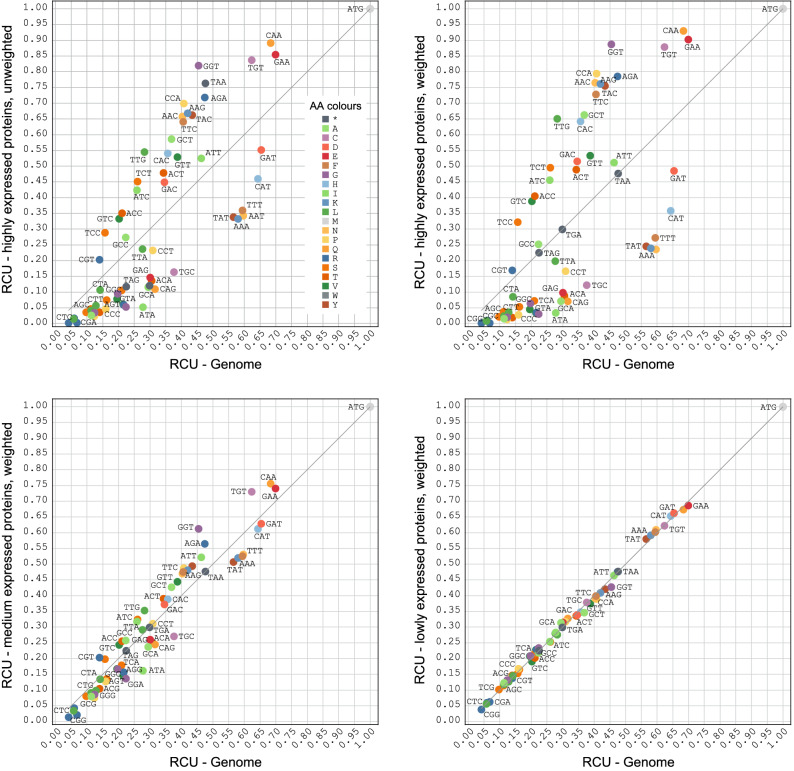
GPome-plots (genome versus proteome plots). The plots show the relative codon usage (RCU) of the 308 most expressed proteins (“highly expressed”), the following 1013 proteins with medium expression, and the 5024 least expressed proteins of S. cerevisiae plotted against the RCU of all predicted yeast genes (x-axis). For comparison, the RCUs of the highly expressed proteins are shown unweighted and weighted. Weighting means that each gene is multiplied by its absolute abundance as given by the PaxDB data.

All these approaches are confined by extrapolation of limited data or assumptions on codon evolution models. To overcome these limitations, we suggest using the term preferred codon only for codons in genes whose protein products have the highest measured abundance in the cell. As a first and rough estimation available DNA microarray data can be used^24^. Even more, quantitative protein abundance data are available at PaxDB for many organisms and can be segmented by absolute or relative criteria. The difference between the codon usage derived from the genome versus that present in a selected proteome can best be visualized in GPome-plots (genome versus proteome plots; Fig. 3), which have been introduced recently^40^. Proteins with the highest abundance in *S. cerevisiae* show the largest codon usage bias, and genes with no detectable translation have a correspondingly inverted codon usage. The analysis also shows that several codons are almost not used at all, similar to the so-called [RIL]-codons in *E. coli* bacteria, but that there is not a single codon box, in which one of the synonymous codons is used exclusively (except for the trivial one-codon one-amino acid boxes). Accordingly, selecting only the preferred codons for heterologous gene design will cause highly biased and atypical codon usage patterns.

The protein abundance does not decrease in steps but exponentially across all proteins (see PaxDB for data). Of course, the abundance is not a function of codon usage alone. In order to reflect the very different abundance across large sets of proteins (e.g. the 50 or 250 most highly expressed proteins), we introduced a weighting scheme. While in the unweighted RCU each codon of each gene in the selected set of proteins is counted once, in the weighted RCU each codon of each gene is multiplied with the abundance of the protein according to the PaxDB data (Fig. 3).

### Typical genes for every purpose

For synthetic biology applications there is not only need for protein production (e.g. highest protein expression) but also for functional studies at expression levels comparable to that of endogenous proteins, or at low levels to avoid toxic effects to name a few. Thus, it would be favourable to generate heterologous DNA sequences for every expression level or expression purpose. We suggest terming such heterologous sequences “typical genes” as they are intended to resemble other genes with a similar expression level. To generate such typical gene sequences we developed Odysseus, which is available online at http://odysseus.motorprotein.de. Its core feature is the adaptation of coding sequences to the characteristics of a pre-selected reference gene set of a host organism to increase or decrease the expression rates of the designed proteins. This is done by using a probabilistic model in form of a Markov chain with the RCU as stationary probabilities and the RSdCU as transition probabilities, both trained with host-specific codon-usage information. Odysseus does not generate a single, perfectly optimized gene but provides multiple genes that are all equally typical with respect to the selected reference gene set. The initial set of typical genes is subsequently filtered by excluding candidate genes containing user-defined features such as unwanted enzyme restriction sites. We think that excluding entire genes (and re-generating more typical genes in case all candidates contain unwanted enzyme restriction sites, for example) is the better solution compared to changing a typical gene sequence (to remove unwanted enzyme restriction sites, for example), because the latter might change the di-codon usage to non-typical patterns. In principle, any additional local feature could be implemented to be filtered at this stage as well, such as patterns resembling Shine-Dalgarno consensus sequences, premature poly(A) translation termination sites, CpG islands, cryptic splice sites, dyad repeat sequences or RNase E cleavage sites. The filtered sequences are subjected to RNA secondary structure prediction and the final set of typical genes is presented in a comparative view. Here, the user can inspect the characteristics of each sequence (e.g. RNA secondary structure, restriction sites) and select sequences for DNA synthesis. For validation of the new approach, we choose a highly structured protein, GFP, and an intrinsically disordered protein, human αSyn.

### Expression of typical genes encoding GFP in *S. cerevisiae*

GFP is a compact, stable beta-barrel forming protein derived from the jellyfish *Aequorea victoria* that can be expressed in almost every organism^41^. Therefore, it is used as a *gold standard* for testing gene design algorithms and analysing protein expression characteristics such as translational efficiency and accuracy. To test the Odysseus algorithm, we generated genes based on the codon usage of the 5024 lowest expressed (abundance-threshold 88.8 ppm) and of the 308 highest expressed proteins (abundance-threshold 663.0 ppm) in *S. cerevisiae* as determined by PaxDB data (dataset 4932-WHOLE_ORGANISM-integrated.txt; weighted average of all *S. cerevisiae* WHOLE_ORGANISM datasets). In both cases we used the weighting scheme as described above.

**Figure 4:**
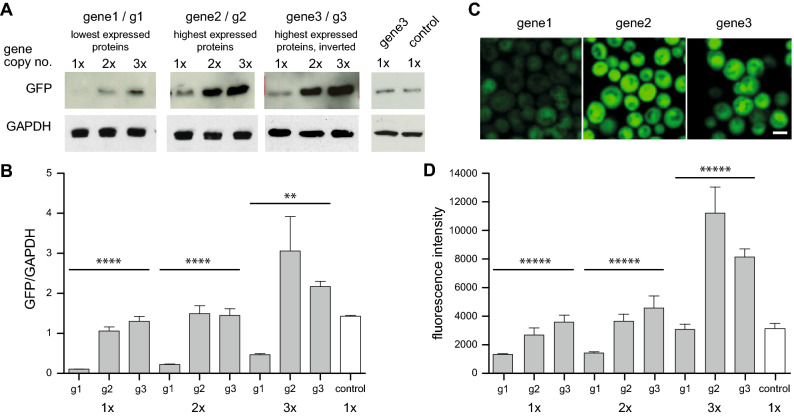
Steady-state protein levels of GFP. Three types of gene design were tested in combination with one to three gene copies. All designed genes are based on the weighting scheme, by which each codon of a subset of genes is multiplied with its expression level as provided by PaxDB data. Gene1 is based on the subset of the 5024 least expressed genes, gene2 is based on the 308 highest expressed genes, and gene3 is based on the inversion of the codon usage of the highest expressed genes. (**A**) Western blot analysis of crude protein extracts from yeast strains, expressing *GAL1-*driven GFP from one, two and three copies. Protein expression was induced for 6 h in galactose-containing medium, crude protein extracts were prepared and equal protein amounts from all samples were used for Western blotting. The membrane was probed with anti-GFP antibody. GAPDH antibody was used as a loading control. The full-sized blots are available in Supplementary Fig. S2. (**B**) Quantification of the protein levels of GFP. Densitometric analysis of the immunodetection of GFP, relative to GAPDH loading control. The significance of the differences was calculated with a One-way Anova-test (**p = 0.002; ****p < 0.0001; n = 3). (**C**) Life-cell fluorescence microscopy of yeast cells, expressing GFP from three copies. Scale bar: 5 µm. (**D**) Quantification of the fluorescence intensity of GFP-expressing cells with different copy numbers and coding sequences. The mean fluorescence intensities were quantified using SlideBook6 software package (n = 100 per strain, except n = 200 for the control). The significance of the differences was calculated with a One-way Anova-test (*****p = 0.0).

For precise comparison of the protein levels, we avoided the typical plasmid-borne expression of the designed genes that might reflect variations in the plasmid copy number in different cell populations. Therefore, yeast strains were generated with genomically integrated either one, two or three copies of the designed GFP-encoding genes, driven by the inducible *GAL1* promoter (Fig. 4; Supplementary Figs. S1 and S2). As control, we analysed a GFP gene without any nucleotide changes. Expression of GFP was induced for 6 h and the protein levels were analysed by Western blot analysis (Fig. 4A,B; Supplementary Fig. S3). The results of these expression test support our initial idea of generating typical genes for typical protein expression ranges. The expression of the gene based on the codon usage of the lowest expressed proteins (gene1) is considerably lower than the expression of the gene based on the codon usage of the highest expressed proteins (gene2). The expression of both proteins considerably increases when increasing genomic copy numbers. The expression level of the control is similar to the expression level of the gene based on the highest expressed proteins. Additionally, live-cell fluorescence microscopy was performed with cells, expressing GFP from different genes. Quantification of the GFP fluorescence intensity corroborated the results from the Western blot analysis and revealed similar differences in the GFP fluorescence depending on the codon context (Fig. 4C,D).

If our experiments represented typical lowly and highly expressed genes, then genes N-terminally fused with GFP-tag would also represent highly or lowly expressed genes, depending on the GFP sequence. The first 30–50 codons at the 5′ end are thought to determine the expression efficiency and protein level (also called ramp sequence)^42^ implying that the expression level of 3′-fused genes will be similar to that of GFP alone. These data might explain the observation that the expression of GFP-fused genes often depends on whether the genes are fused to the 5′ or 3′ end. Instead of supposed folding problems of the fused proteins, the difference in expression level might mainly depend on whether the fused gene resembles a typical lowly of highly expressed gene. Our experiments suggest that the designed GFP resembling lowly expressed genes could be used for studies of cellular protein expression if the expression level needs to resemble endogenous low levels.

### Expression of human α-synuclein in *S. cerevisiae*

**Figure 5:**
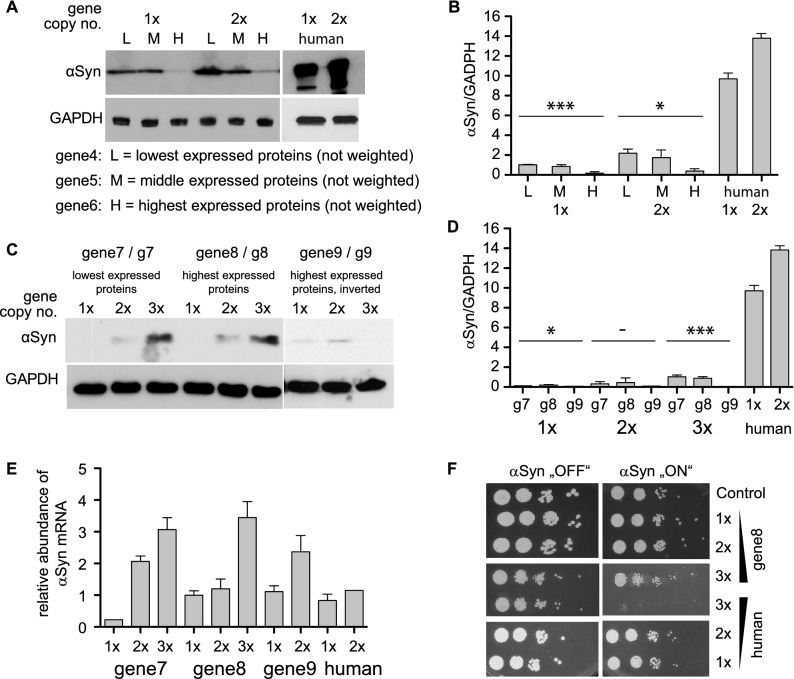
Expression of designed and human α-synuclein. (**A**) Western blot analysis for determination of the protein level of αSyn. Protein expression was induced for 6 h, crude protein extracts were prepared and the protein concentrations were determined with a Bradford assay. 160 μg crude protein extract from samples gene4 (L), gene5 (M) and gene6 (H), and 40 μg from samples “human” were used for Western blotting. The membrane was probed with anti αSyn antibody. GAPDH antibody was used as a loading control. The full-sized blots are available in Supplementary Fig. S3. (**B**) Quantification of the protein levels of αSyn. Densitometric analysis of the immunodetection of αSyn, relative to GAPDH loading control. The significance of the differences was calculated with a One-way Anova-test (*p = 0.0107; ***p = 0.00014; n = 3). (**C**) Western blot analysis of crude protein extracts from yeast strains, expressing *GAL1-*driven αSyn from one, two and three copies. Protein expression was induced for 6 h, crude protein extracts were prepared and the protein concentrations were determined with a Bradford assay. 160 μg crude protein extract from samples gene7, gene8 and gene9, and 40 μg from samples “human” were used for Western blotting. The membrane was probed with anti-αSyn antibody. GAPDH antibody was used as a loading control. The full-sized blots are available in Supplementary Fig. S4. (**D**) Quantification of the steady-state protein level of αSyn. Densitometric analysis of the immunodetection of αSyn, relative to GAPDH loading control. The significance of the differences was calculated with a One-way Anova-test (*p = 0.038; -p = 0.41; ***p = 0.00034; n = 3). (**E**) Quantification of *SNCA* gene expression. RNA was prepared from yeast strains after 6 h induction of αSyn expression. Relative αSyn mRNA levels were determined by qRT-PCR and normalized against H2A. Expression values represent the mean of three replicates ± standard error. (**F**) Growth analysis of yeast cells expressing αSyn from one, two and three gene copies, driven by the inducible *GAL1-*promoter on non-inducing (‘OFF’: glucose) and inducing (‘ON’: galactose) SC-URA medium after 3 days. Yeast cells expressing GFP from the same promoter were used as a control.

Intrinsically disordered proteins play important function in cellular signalling and regulation pathways^43^. As a test case for an intrinsically disordered protein, we choose human αSyn. The protein αSyn has a central role in the pathogenesis of Parkinson's disease (PD). Accumulation of this highly soluble protein leads to aggregation and proteotoxicity in several neurodegenerative diseases^44^. Expression of human αSyn in yeast faithfully reproduces the molecular mechanisms that results in aggregation and cellular toxicity^45,46^. Importantly, the toxicity is dose-dependent and directly correlates with αSyn expression level^32,47^. We used the advantages of this humanized yeast model, where the toxic effects depend on αSyn gene expression and assessed, whether the toxicity can be rescued by codon adaptation. First, we designed three genes based on subsets of the 5024 lowest expressed proteins in yeast (according to PaxDB; gene4), 1013 proteins with medium expression level (gene5), and the 308 highest expressed proteins (gene6; Fig. 5). As reference we expressed αSyn from the human coding *SNCA* gene sequence. Yeast strains were generated with genomically integrated one or two copies of the designed αSyn-encoding genes, driven by *GAL1* promoter (Supplementary Fig. S1). Expression of αSyn was induced for 6 h and the protein levels were analysed by Western blot analysis (Fig. 5A,B). Surprisingly, the designed genes showed considerably lower expression compared to the human reference, although their codon composition had been adapted to the yeast host organism. Even more surprisingly, the expression level decreased from the gene based on the lowest expressed proteins to the gene based on the highest expressed proteins. This indicates that adaptation of the codon usage of a gene of interest to the highest expressed proteins of a species does not always yield highest expression, which is consistent with observations of researchers trying to boost expression levels, e.g. for structural biology.

### Expression of human α-synuclein in *S. cerevisiae* using weighted codon usages

To exclude that our observation depends on not having used the weighting of the protein abundance levels, we designed genes based on the lowest and highest expressed proteins, respectively, using the weighting scheme (gene7 and gene8, respectively; Fig. 5C,D; Supplementary Fig. S4). Again, for precise comparison of αSyn protein levels strains with one, two or three copies of the designed genes were generated. Western blot analysis revealed significantly lower protein levels than the human reference gene, similar to the results with genes 4–6. Next, we assessed whether the differences in αSyn expression level are mediated by an impact of the codon usage on transcription. Comparison of the mRNA levels in these strains showed similar gene expression of the designed genes and the human gene (Fig. 5E). This suggest that the effect of different codon usage for αSyn is mainly due to its impacts on translation.

### Expression of GFP and α-synuclein using an inverted codon usage pattern

**Figure 6:**
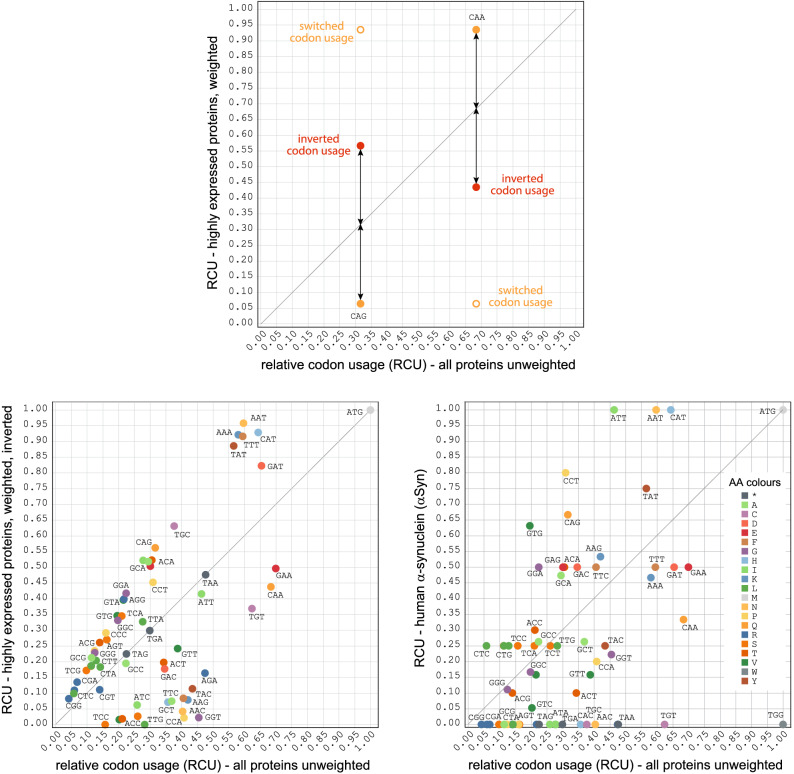
The “inverted” codon usage. The schematic view at the top demonstrates the generation of an inverted codon usage compared to that of a switched codon usage. The plots at the bottom show the relative codon usage of the 308 highest expressed proteins, when weighted and inverted (left plot), and the relative codon usage of human αSyn (right plot).

To identify potential abnormalities within the human reference αSyn gene with respect to the designed yeast genes, we compared their codon usage. The human reference gene does not include many of the codons, which are preferentially used in the highest expressed genes, nor does it include many of the rare codons (Fig. 6). Instead, it appears that the human αSyn gene resembles an inverted codon usage scheme of the highest expressed yeast genes. With inverted scheme it is not meant that the codon usage is switched (e.g. if the codon usage of CAA and CAG were 0.93 and 0.17, respectively, switching would mean 0.17 and 0.93 for CAA and CAG; Fig. 6), but that the codon usage is inverted at the values of the genomic codon usage (e.g. if the genomic codon usage of CAA and CAG were 0.68 and 0.32 and the codon usage of these codons across the highest expressed genes were 0.93 and 0.07, respectively, inverting the codon usage would result in codon usage of 0.43 and 0.57 for CAA and CAG, respectively; Fig. 6; Supplementary Fig. S5). To test whether genes designed with such an inverted codon usage scheme still resemble typical genes, we designed and tested a GFP gene with inverted codon usage (gene3; Fig. 4). The protein expression level of this GFP gene rather resembled that of the gene based on the highest expressed yeast proteins than that of the gene based on the lowest expressed proteins. The designed αSyn gene based on the inverted codon usage did not result in higher expression, however, compared to the genes without inverting the codon usage (Fig. 5C,D). These two test cases demonstrate that genes based on an inverted codon usage can well be expressed. This way rare codons are used more often, although not exclusively. The comparable expression levels of GFP based on the inverted and the highly expressed genes imply that tRNA abundance, which is commonly assumed to be lower for the rare codons, does not determine expression level. However, more tests with more types of genes are needed to fully assess, in which cases the inverted codon usage pattern might be preferable to other patterns.

Finally, we assessed whether the observed low protein levels affect αSyn induced toxicity, reflected as growth retardation. Growth assays were performed with αSyn, expressed from different gene copy numbers and codon context. Expression of αSyn from three copies of gene8 did not reveal toxic phenotype in contrast to its human counterpart that is toxic in yeast (Fig. 5F)^32^. These results demonstrate that we could successfully implement the newly developed design algorithm for reducing the expression level of a toxic protein in yeast, exemplified by the use of αSyn.

### Comparison to other software

There are multiple services for adapting gene sequences to heterologous hosts or for designing gene sequences from scratch. For example, the TISIGNER software has been developed to adjust translation initiation sites by optimizing mRNA accessibility (reducing mRNA secondary structures) and can thus be used to optimize the 5′-end of a gene^48^. TISIGNER requires at least part of the 5′-UTR as input in addition to the gene sequence. As discussed above, most software to design entire gene sequences optimize the CAI. However, the used codon usage tables most often do not refer to the codon usage of only the highly expressed genes but that of all genes, which rather resembles the usage of the lowly expressed genes. For example, the Gene Designer v1 software (the algorithm of v2 is not published and could be different) uses the frequency distribution for each codon box based on a codon usage table, which according to the online documentation does not correspond to the usage of the highly expressed genes in yeast^49^. The tool OPTIMIZER allows the upload of a user-provided codon usage table and selection of always the most used codon, random selection by frequency distribution or manual selection of codons^50^. In a very recent approach, ChimeraUGEM, a target gene is designed by comparing its protein sequences by a longest substring approach to a set of reference sequences assuming that longer repetitive substrings became more optimized to the hosts translation machinery^51^. The algorithm has been used for predicting gene expression levels, and has also been shown to be successful in increasing expression of a synthetic gene in green algae. The ChimeraUGEM approach seems to be most related to our typical gene approach, although ChimeraUGEM designs the genes substring by substring (not overlapping) and does not explicitly aim to optimize di-codons.

### Limitations of the odysseus implementation

It is well known that there is additional information in the coding sequence of a gene beyond the genetic code for translating nucleotide triplets (codons). For example, the UGA stop codon is translated to selenocysteine if a so-called SECIS pattern is present^52,53^. There is also a process termed programmed ribosomal frameshifting by which the ribosome shifts the reading frame by one or two nucleotides in either the + or the − direction^54^. The patterns of these two examples, selenocysteine decoding and ribosomal frameshifting, dictate the protein sequence. These patterns are not implemented yet. The Odysseus tool does also not allow to adjust the gene sequence to other genetic codes than the standard genetic code which could be a useful extension. It is recommended that users select one of those suggested typical genes, that do not contain the reassigned codon (e.g. does not contain a CTG codon if protein expression in *Candida albicans* is wanted). However, the designed genes might not be typical anymore in species that heavily use the reassigned codons such as several ciliates, that decode stop codons by glutamine^55^. In addition to codes leading to different protein sequences, there are also various regulatory signals on top of the coding region that affect the gene expression and protein translation levels^56^. Odysseus is not aware of these signals and the designed genes might miss important signals (if wanted) or by chance introduce unwanted signals. As far as those signals or sequence patterns are known a user could manually detect this and select another of the set of typical genes that Odysseus generates.

## Conclusions

Odysseus is a new software tool to design typical genes for heterologous protein expression. In contrast to most other tools, which intend to optimize the codon usage by selecting only codons from a few highly expressed proteins or by selecting only the codon with the highest relative codon usage from each codon box, Odysseus generates genes resembling the codon usage of a selected group of proteins. Such groups can be the highest or lowest expressed proteins of a species (with the cut-off free to choose), or even a subset of proteins with a certain function. We tested the new system by generating synthetic genes of the non-toxic, highly structured protein GFP and by evaluating their expression level. The expression level strongly increased from the gene based on the lowest expressed proteins to the gene based on the highest expressed proteins. This supports the general finding that protein expression is stronger when adapting a heterologous gene to the most used codons. Such a strong expression is, however, often not wanted and disfavoured when trying to express a toxic protein. To test our software for its use for expressing proteins at low endogenous protein expression levels, we designed synthetic genes for the toxic, non-structured protein αSyn and showed that human αSyn can be adapted to low expression levels. Although further tests with more proteins are needed our results suggest that Odysseus is a valuable tool for designing typical genes for heterologous protein expression.

## Supplementary Information

Supplementary Figures.

## Data Availability

The software can be used via a web interface at http://odysseus.motorprotein.de, and obtained from GitHub at https://github.com/dsimm/Odysseus for local installation and use.
