# Supplementary material for: Design of typical genes for heterologous gene expression
Source: Sci Rep. 2022 Jun 10;12:9625. doi: 10.1038/s41598-022-13089-1 (PMC9187722; doi:10.1038/s41598-022-13089-1)
Supplement: Supplementary file 1 — Supplementary Figures. [file 41598_2022_13089_MOESM1_ESM.pdf]

Supplementary Figures

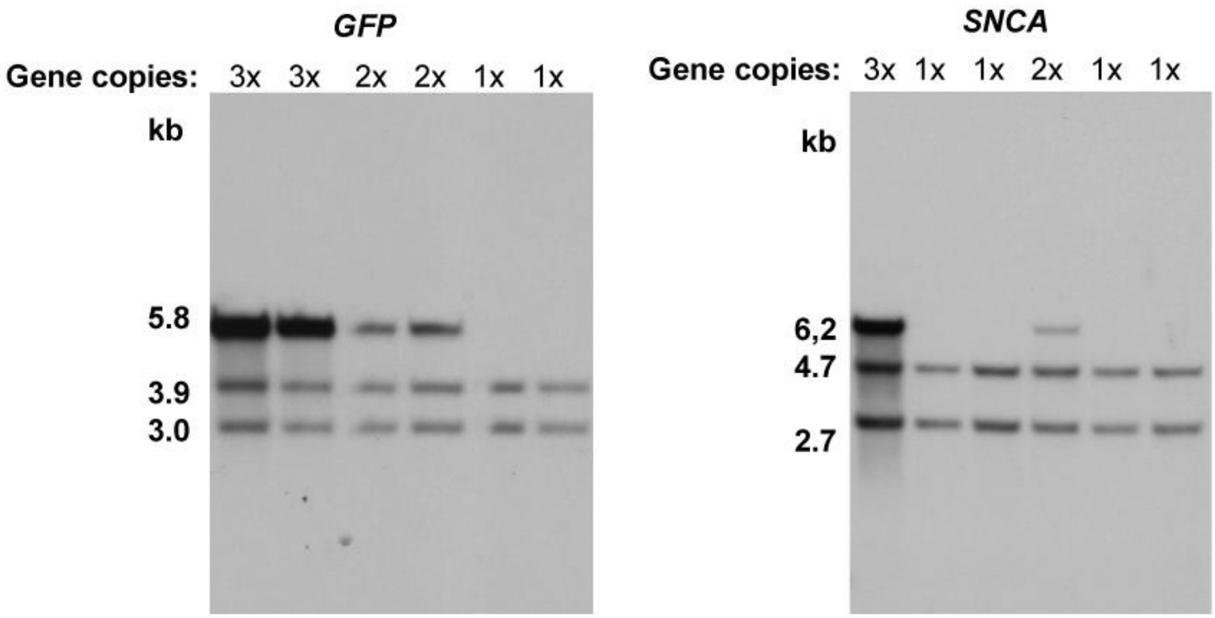

Figure S1: Southern hybridization and copy number determination. Yeast cells were transformed with integrative plasmids, harboring *GFP*- or αSyn-encoding genes, respectively. Multiple transformants were analyzed by Southern hybridization for verification of the integration of *GFP* or αSyn genes into the mutated genomic ura3-52 locus using labeled URA3 as a probe. Left plots: One copy (1x) of integrated *GFP* corresponds to 3.0 kb + 3.9 kb; two copies (2x) to 3.0 kb + 3.9 kb + 5.8 kb, and three copies (3x) to 3.0 kb + 3.9 kb + 5.8 kb (higher intensity). Right plots: One copy (1x) of integrated αSyn corresponds to 2.7 kb + 4.7 kb; two copies (2x) to 2.7 kb + 4.7 kb + 6.2 kb, and three copies (3x) to 2.7 kb + 4.7 kb + 6.2 kb (higher intensity), as indicated. Copy numbers were determined with the ImageJ software.

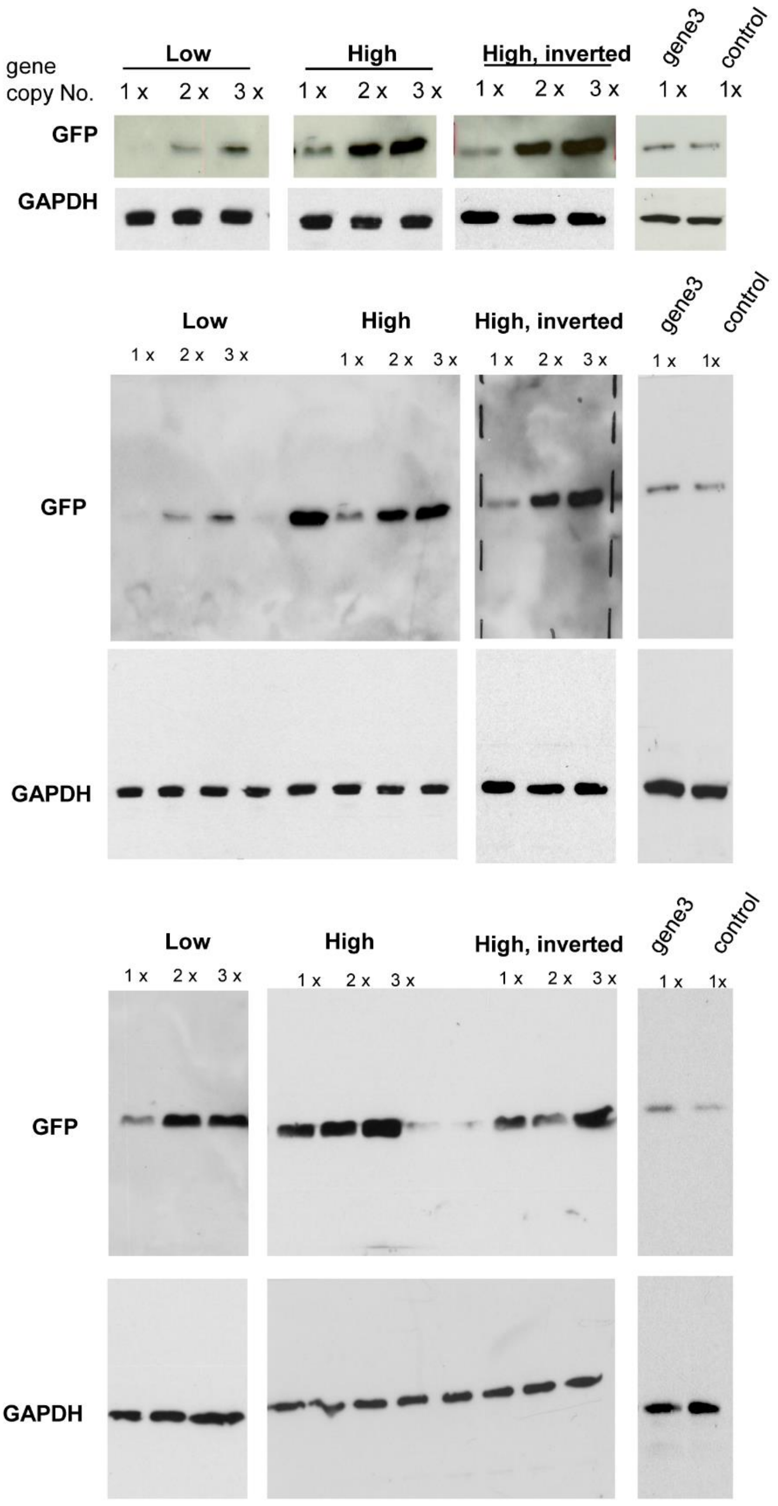

Figure S2: Full size blots corresponding to Figure 4A. On top, the part shown in Figure 4A is presented for comparison. Below, the blots of two replicates are shown. Western blot analysis of crude protein extracts from yeast strains, expressing *GAL1-*

driven GFP from one, two and three copies. Protein expression was induced for 6 h in galactose-containing medium, crude protein extracts were prepared and equal protein amounts from all samples were used for Western blotting. The membrane was probed with anti-GFP antibody. GAPDH antibody was used as a loading control.

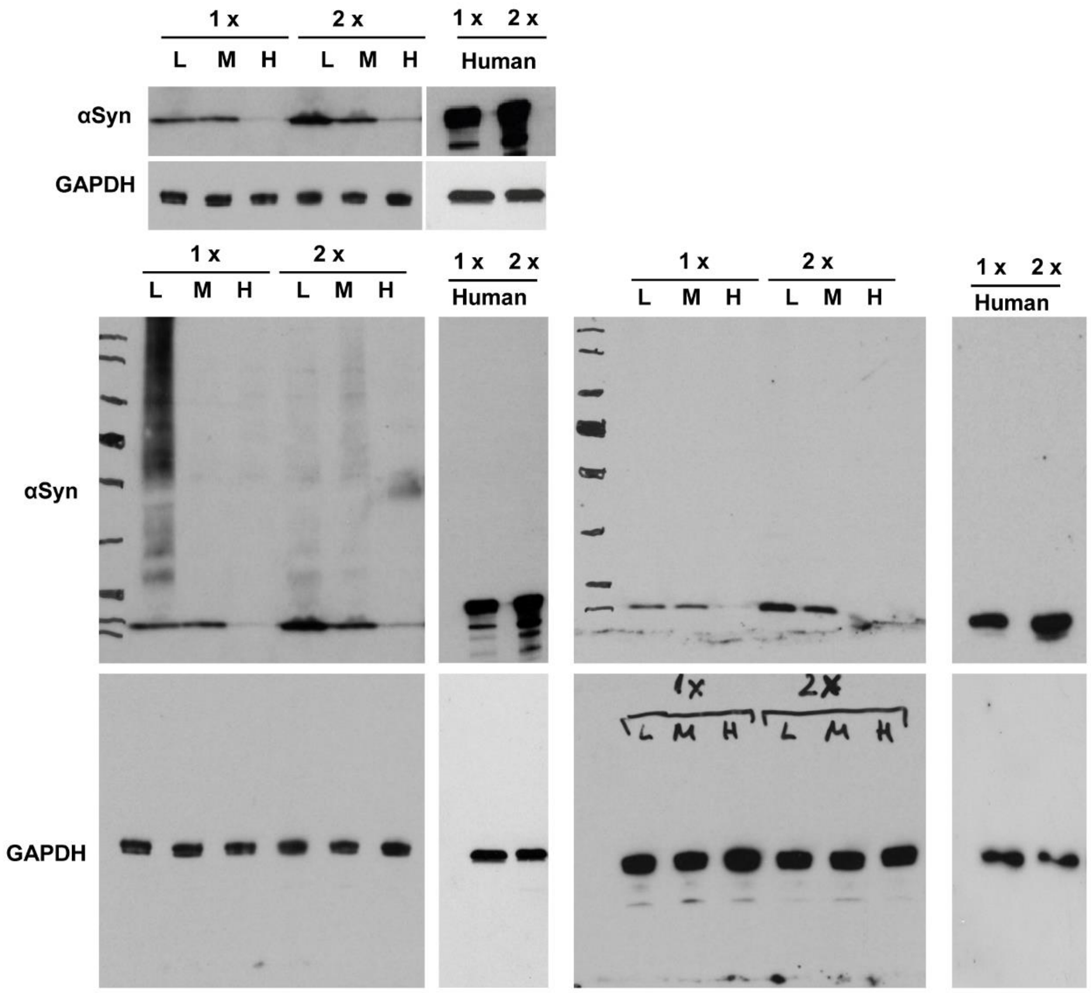

Figure S3: Full size blots corresponding to Figure 5A. On top, the part shown in Figure 5A is presented for comparison. Below, the blots of two replicates are shown. Western blot analysis for determination of the protein level of αSyn. Protein expression was induced for 6 h, crude protein extracts were prepared and the protein concentrations were determined with a Bradford assay. 160 µg crude protein extract from samples gene4 (L), gene5 (M) and gene6 (H), and 40 µg from samples “human” were used for Western blotting. The membrane was probed with anti αSyn antibody. GAPDH antibody was used as a loading control.

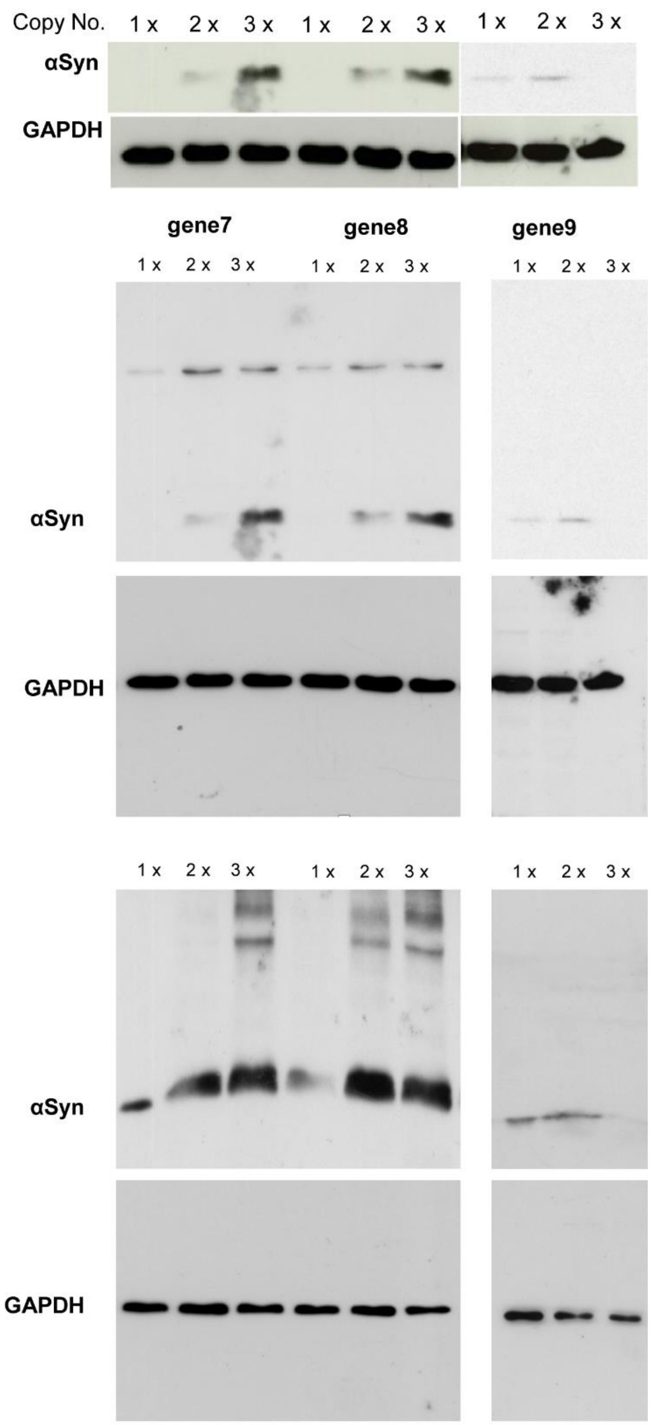

Figure S4: Full size blots corresponding to Figure 5C. On top, the part shown in Figure 5C is presented for comparison. Below, the blots of two replicates are shown. Western blot analysis of crude protein extracts from yeast strains, expressing *GAL1*-driven αSyn from one, two and three copies. Protein expression was induced for 6 h, crude protein extracts were prepared and the protein concentrations were determined

with a Bradford assay. 160 µg crude protein extract from samples gene7, gene8 and gene9, and 40 µg from samples “human” were used for Western blotting. The membrane was probed with anti-αSyn antibody. GAPDH antibody was used as a loading control.

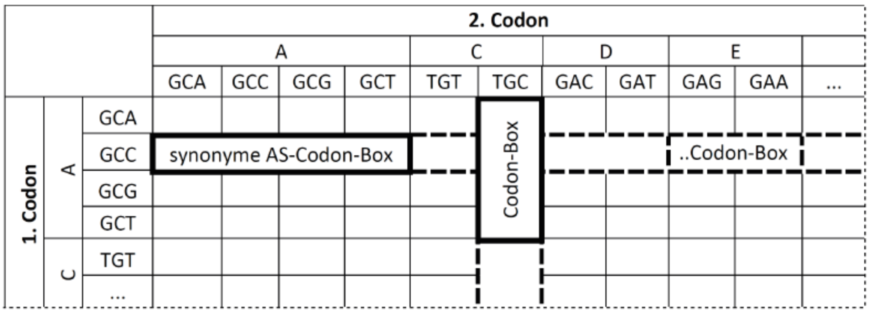
2. Codon
A
C
D
E
GCA
GCC
GCG
GCT
TGT
TGC
GAC
GAT
GAG
GAA
...
1. Codon
A
C
GCA
GCC
GCG
GCT
TGT
...
synonyme AS-Codon-Box
Codon-Box
..Codon-Box

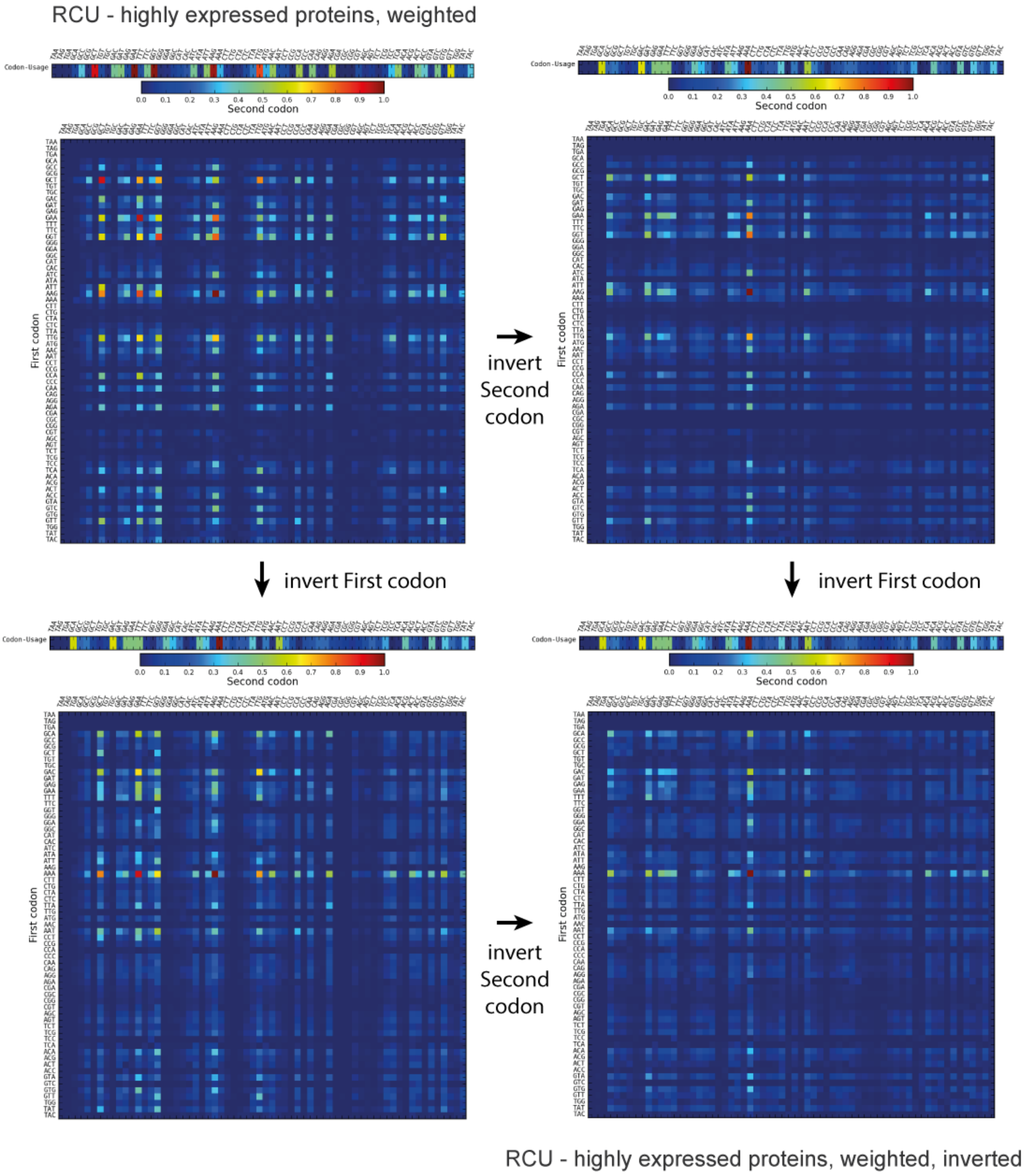
RCU - highly expressed proteins, weighted
Codon-Usage
Second codon
First codon
invert Second codon
invert First codon
invert First codon
invert Second codon
RCU - highly expressed proteins, weighted, inverted

Figure S5: The “inverted” di-codon usage. The schematic view at the top shows example codon boxes within the di-codon usage matrix. The heatmap plots at the bottom show the relative codon usage of the 308 highest expressed proteins of *S. cerevisiae*, when weighted (top-left), and the two ways to get the di-codon usage inverted (bottom-right).
